# Solving the “General Elution Problem” of Ion Mobility Spectrometry: Single-Run Voltage Sweep

**DOI:** 10.1021/acs.analchem.5c04496

**Published:** 2025-11-10

**Authors:** Addison Bale, Tristan Koop, Landon Vyhmeister, Gavin Valdez, Julia Fehr, Eric Davis

**Affiliations:** Department of Chemistry, Whitworth University, Spokane, Washington 99251, United States

## Abstract

While modern mobility methods have shifted toward vacuum-based techniques that allow advanced ion manipulations to improve resolution, atmospheric pressure ion mobility spectrometry (IMS) cells continue to find utility in primary security and screening applications. These small units generally demonstrate low resolution due to both their size and inability to perform advanced ion manipulations at atmospheric pressure. Existing drift time IMS cells utilize a static voltage typically tuned to optimize both total signal and separation efficiency for the ions of particular interest in a given application. These static voltage IMS cells suffer from a phenomenon similar to the general elution problem of chromatography, wherein the separation conditions are optimized for a particular species at the expense of the other analytes in the system. Through the application of an external high voltage sweep (VS) tuned to match the theoretical optimum potential at every drift time, this work solves the General Elution Problem of IMS. In comparison to static mode, VSIMS demonstrates significantly improved resolution and resolving power while maintaining fidelity in reduced mobility measurements. When applied to a library of IMS standards, VSIMS demonstrates consistent, reproducible resolving powers regardless of the mobility of the ion introduced to the instrument.

## Graphical abstract

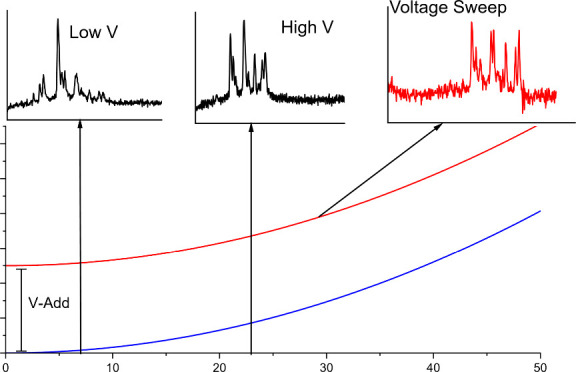

## Introduction

Drift Tube Ion Mobility Spectrometry (DTIMS) provides size-to-charge ratio data complementary to mass spectrometry measurements for many applications. IMS is most commonly applied in security checkpoints as a primary screening method for illicit materials or explosives.^1–5^ However, while a mature technology, its scientific applications are limited by its inherent low resolution. Most drift-tube IMS cells produce a resolving power $\left(\frac{time}{FWHM}\right)$ from 40 to 70 ^6,7^ in comparison to the 1000+ common for routine mass-spectral measurements.^8^ Modern methods for addressing this technology gap have focused on low-pressure IMS measurements where advanced ion manipulation becomes possible. These include cyclic IMS, wherein an ion packet may be maintained in a circular loop, indefinitely increasing the apparent length of the drift cell and providing extremely high-resolution measurements.^9–11^ In a similar experiment, Structures for Lossless Ion Manipulation (SLIM) IMS allows a similar improvement in resolution through repetitive cycling of ions under vacuum conditions.^12–17^

Portable IMS cells common to security or military applications have not adopted vacuum-based methods due to the increase in maintenance, cost, and decreased portability inherent with high vacuum systems. Thus, these separations rely upon traditional, gated drift time measurements and are thereby relegated to primary screen due to their low resolution. DTIMS also allow the direct calculation of collision cross section (CCS)^6^ values without the external calibration required by the traveling wave technology^18,19^ on which both cyclic and SLIM IMS methods rely.

Prior work demonstrates a relationship between the mobility of an ion and the optimal electric field under which that ion is best separated.^20,21^ Through the application of an externally changed electric field, stepped through the optimal mobility range of an instrument, this work demonstrated that this mobility dependence resulted in a phenomenon similar to the “general elution problem” of chromatography.^8^ However, this work depended on the slow process of stepping the potential through a wide range, taking full DTIMS spectra at each value, before producing an optimized spectrum in postprocessing of the data. This postprocessing produced an optimized spectrum wherein the electric field was effectively manipulated during the analysis so that each ion was separated under optimal conditions. However, this experiment was extremely slow (minutes to hours), negating the use of this technique in high-throughput applications.

More recent work by Reinecke et al. demonstrated a unique combination of a modified electric field coupled with a multiplexing experiment in DTIMS.^22^ In this work, rather than sweeping the frequency relationship between the first and second gates in the IMS cell, the potential on the cell was swept over a period of several seconds while the gating frequency was held constant. This work provided a simple mechanism for interfacing an IMS to a slower technique, such as mass spectrometry, while maintaining fidelity of the mobility data. However, the resultant data demonstrated a decrease in resolving power, counter to that demonstrated in the stepped voltage sweep experiments previously mentioned.

With the modern advent of high voltage amplifiers capable of reproducibly generating a high voltage sweep with a slope greater than 500 V/μs, the culmination of this prior work may now be realized. By applying the theoretical optimum potential to the IMS cell at any given time in the separation, an IMS cell may be effectively operated under ideal conditions for all ions, thus solving the general elution problem inherent in this separation technique.

## Theory

Reduced Mobility (K_0_) is a fundamental concept in IMS related to the collision cross section of an ion:^6^

$$K_{0}=\frac{L^{2}}{V^{*}t_{d}}\frac{P}{760}\frac{273.15}{T}$$ 1

Resolving power in an ion mobility experiment has been established as a relationship between the width of the ion packet input to the separation (gate pulse width) and simple diffusion of the ion packet as the ions transit the drift region:^23–25^

$$R_{c}=\frac{1}{\sqrt{{\left(\frac{760}{273.15}\right)}^{2}\frac{t_{g}^{2}K_{0}^{2}T^{2}V^{2}}{L^{4}P^{2}}+\frac{16k_{B}T\ln2}{qV}}}$$ 2

Where t_g_ is the pulse width of the ion gate (in seconds), K_0_ is the reduced mobility of the ion of interest, T is the temperature (in K), V is the potential drop across the drift space, L is the length of the drift region, P is the pressure of the system (in Torr), k_B_ is the Boltzmann Constant, and q is the elementary charge. This equation results in a maximum resolving power for any given compound (K_0_) dependent on the characteristics of the drift cell used and the ambient conditions that can be derivatized to an optimum voltage applied to the drift cell for a given compound:^20,21^

$$V_{opt}=0.0395{\left[\frac{L^{4}P^{2}}{t_{g}^{2}K_{0}^{2}T}\right]}^{1/3}$$ 3

By substituting the definition of reduced mobility into this equation, the optimum potential is found to be dependent only on the temperature, gate pulse width, and drift time of an ion to calculate the optimum voltage with respect to drift time:

$$V_{opt}={\left(t_{d}\frac{760}{273.15}\frac{0.0395T}{t_{g}}\right)}^{2}\frac{0.0395}{T}$$ 4

This results in an optimum voltage vs drift time curve (Figure 1 – blue trace, plotted as electric field $\left(\frac{V}{cm}\right)$ for a 10 cm drift cell) which may be applied to an IMS cell with a high voltage amplifier. As this curve begins at a potential of zero, ion gating is impossible due to no movement and the predominance of diffusion at this potential. Therefore, an adjustable voltage was added to this curve to allow ion movement in the low potential (fast drift time) regime and to increase overall ion signal by sacrificing some resolving power away from the ideal. Figure 1 red trace indicates the same curve with the V_add_ potential applied:

$$V_{opt}={\left(t_{d}\frac{760}{273.15}\frac{0.0395T}{t_{g}}\right)}^{2}\frac{0.0395}{T}+V_{add}$$ 5

**Figure 1:**
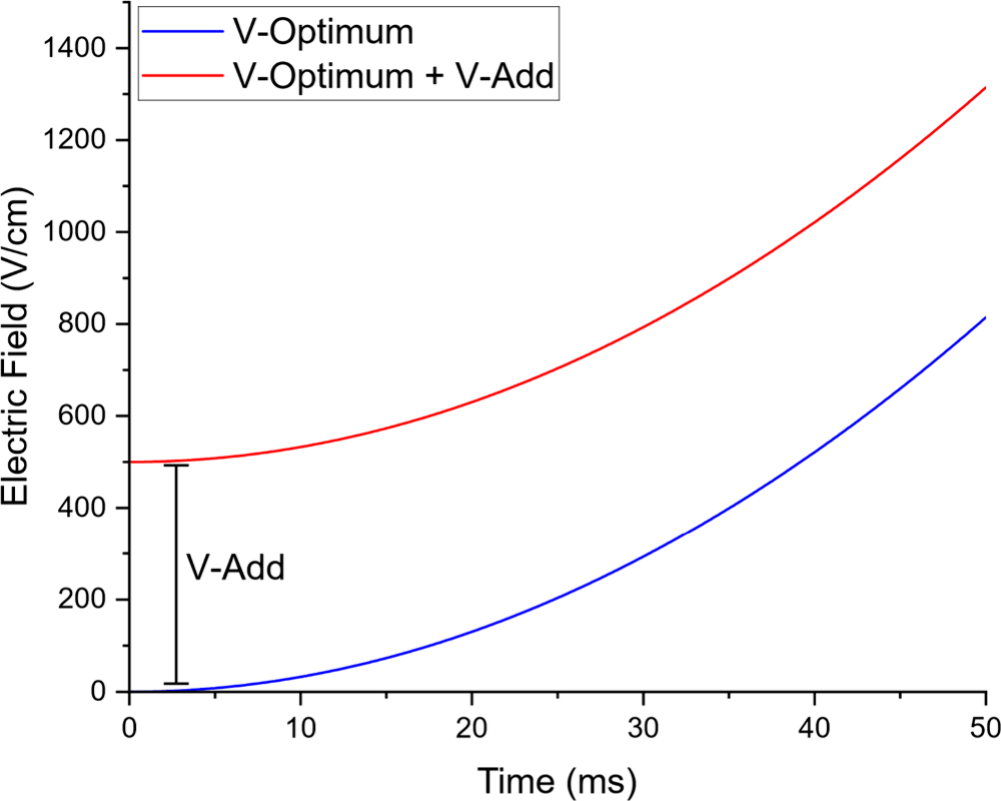
Theoretical potential (blue trace) calculated by eq 4. A direct, exponential relationship between drift time and applied potential is noted. However, to allow ion movement in an IMS cell, an additional potential (V-Add, red trace) must be applied to the IMS cell to achieve ion movement and avoid diffusion losses.

Unfortunately, the use of an ion gate provides a unique challenge with this experiment. In order to achieve maximal resolving powers per the theory presented in Equation [[Disp-formula eq5]], the initial electric field (V_add_/length) is ideally held at the lowest possible value. However, for a mixture of ions, this results in increasingly inefficient gating for larger ions as the applied potential is moved closer to optimum. In initial experiments, it was noted that below a minimum value for V_add_, total ion signal decreased dramatically. Therefore, a minimum electric field of 150 V/cm was used throughout the experiments demonstrated herein.

The application of this voltage curve to the IMS experiment allows every ion to be separated at an optimal voltage. However, it also places the IMS cell into a changing potential regime, negating the capability to calculate the K_0_ of an ion using equation [[Disp-formula eq1]] as the potential is no longer static. Through integrating equation [[Disp-formula eq4]] and dividing by the drift time (number of points), a static potential may be calculated that approximates the potential an ion experienced and allows direct calculation of reduced mobility:

$$V_{avg}=\frac{t_{d}^{2}}{3}{\left(\frac{760}{273.15}\frac{0.0395\times T}{t_{g}^{2}}\right)}^{2}\frac{0.0395}{T}+V_{add}$$ 6

## Experimental Section

### Solutions and Gases

All standards measured were purchased as neat compounds from Sigma-Aldrich (St. Louis, MO). Methamphetamine (Meth), 3, 4-Methylenedioxymethamphetamine (MDMA), 3,6-diacetylmorphine (Heroin), Benzoylmethylecgonine (Cocaine), and Dihydro-14-hyrdoxycodeinone (Oxycodone) were purchased as 1000 ppm solutions in acetonitrile also purchased from Sigma-Aldrich. All solutions were diluted to 10 ppm in HPLC grade 50/50 methanol/water for analysis.

### Instrument

An IMS cell was constructed per Smith et al. and Schramm et. al^26,27^ consisting of two equal, 10 cm halves in a 3D printed housing with the resistor chain held outside the system and coated in high voltage varnish (MG Chemicals 4226 – “Super Corona Dope”) to avoid arcing during the high voltage sweeps applied. Unlike typical IMS cells, no capacitors were used on the system to reduce noise as extra capacitance would preclude the ability to apply the desired high voltage sweep per equation [[Disp-formula eq5]]. A Faraday plate detector was constructed as described by Reinecke et al.^28^ Clean, dry laboratory air was used as the drift gas at a flow rate of 1.0 L/min at atmospheric pressure (∼705 Torr in Spokane, WA) and room temperature (∼20 °C). All samples were introduced via Electrospray Ionization (ESI) with a 3 kV bias above the first ring (swept with the IMS cell to avoid changes in the reaction region ion plume) using a 10 μm glass capillary with a flow rate of 4 μL/min. A custom gate control, previously described,^29^ provided high-resolution gate pulses through a trigrid ion gate previously described.^28,30^ A pair of Trek 20/20C high voltage amplifiers provided static and swept potentials throughout the experiments described, and a Keithley 427 current to voltage amplifier was used for signal amplification. A National Instruments (Austin, TX) PCI-6351 with external BNC-2120 interfaces provided Data Acquisition and Control (DAQ) for gate pulsing, sweep generation, and analog-digital conversions. In voltage sweep mode, an external power supply provided 140 V to the aperture grid immediately preceding the Faraday plate to minimize effects of the changing electric field on the resultant spectrum.

### Software

A custom Python script using the NIDAQmx library and tkinter graphical user interface library provided for all instrumental control, Analog-Digital Conversion, signal averaging, and calculation of the voltage sweep parameters (see Supporting Information). The software allowed the instrument to operate in either static (single voltage throughout the analysis) and Voltage Sweep (VS) modes for direct comparison using the same instrument. All IMS potentials were controlled via this software, with the ionization source potential set as a bias above the first ring. A user control allowed calculation of the potential at the ion gate through a multiplier that was measured for the IMS unit used. For calculation of the VS parameters, it was necessary for the user to input the pressure (in Torr) and temperature (in K) before each experiment as variables in Equation [[Disp-formula eq4]]. A user-adjustable delay period was also included in the software to allow the instrument to stabilize at the initial potential prior to the next VS experiment. The necessity of this delay is discussed below and was optimized to 2.5 s for the instrument used in this study. All spectra were obtained using 500 signal averages for clarity in signal.

### Data Processing

All spectra were processed using Origin 2025 (Northampton, MA). Static mode IMS spectra were normalized before peak picking to determine *fwhm*. VS mode IMS spectra were adjusted for baseline (see Supporting Information Figure S.1 for an example raw VSIMS spectrum) before normalization and peak picking.

## Results and Discussion

### Application of Voltage Sweep to IMS Cell

Due to concerns regarding parasitic capacitance of the IMS cell and associated electronics, a voltage sweep potential curve was applied to the IMS utilized in this experiment while monitored at the first ring and at the gate with an oscilloscope and high voltage oscilloscope probe. Figure 2 demonstrates the gate pulse (blue), theoretical (black), and measured (red) potential for each location. At the front of the cell, no difference was noted between the called and measured potentials. At the gate, however, some deviation is noted, especially as the voltage was rapidly returned to baseline before initiating the next experiment. As this occurred after data collection stopped, it did not affect the mobility experiment. There was a slight (<3%) deviation in the curve up to the maximum resulting in a potential slightly lower than called as the curve swept up. As this experiment started above 0 V (to allow ion gating) and was therefore not at the optimum potential, this slight decrease in the potential that matched the desired curve shape did not have a noticeable effect on the separation experiment beyond requiring a slight modification to the V_add_ potential in equation [[Disp-formula eq6]] when calculating mobility values. For both measurements, the flattening at the high end of the called potentials corresponded to the maximum output of the high voltage amplifiers, thus stopping the ability to sweep the potentials further. Operating parameters were set to avoid ion elution after this point in the sweep. It was found that operation in VS mode required a long (2.5 s) delay time between gate pulses to initiate the next voltage sweep on the IMS cell. If this value was too short, inverse IMS^31,32^ behavior was observed in the ion swarm. It is suspected that internal capacitances in the gate driver hardware prevented normal operation of this device at higher frequencies.

**Figure 2:**
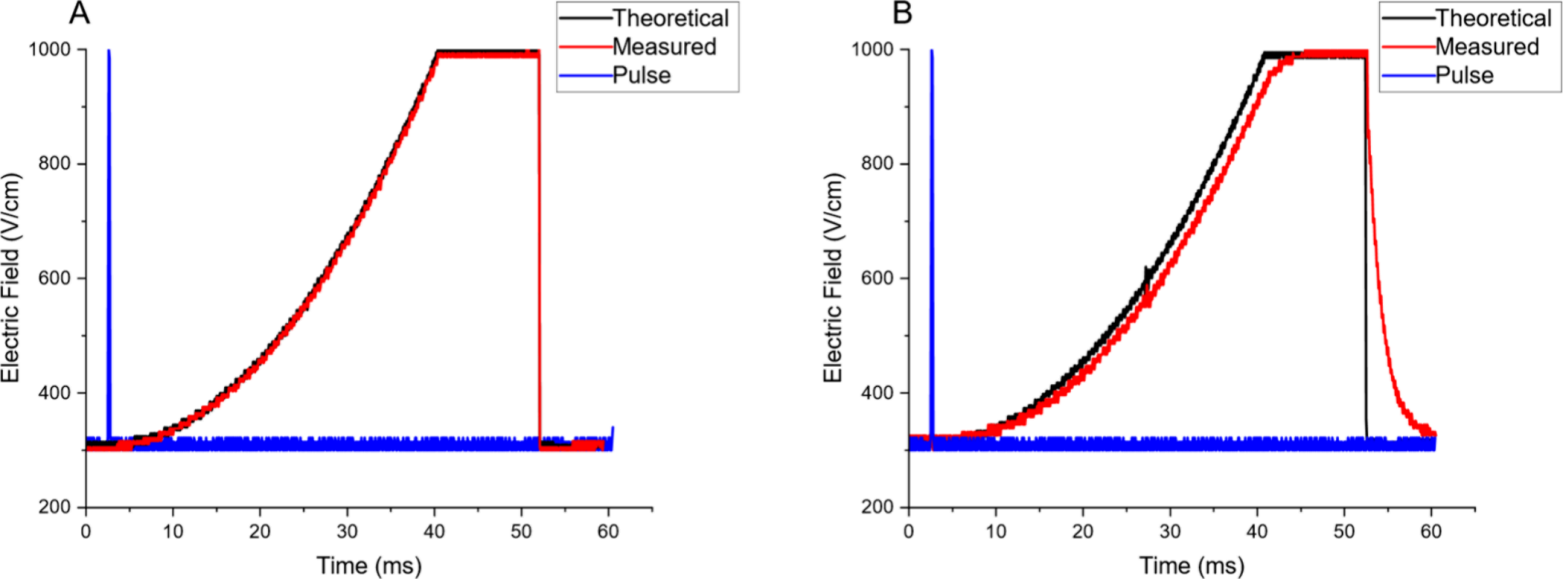
Figure demonstrates the gate pulse (blue), theoretical (black), and measured (red) potential at the first ring (A) and trigrid ion gate (B). All voltage sweeps were analyzed from 300 to 1000 V/cm applied to the first ring.

### Improved Efficiency and Resolving Power

Example spectra obtained in both voltage sweep and static mode are presented in Figure 3 as the first demonstration of the operation of VSIMS. In this figure, a single tetraalkylammonium salt (tetrabutylammonium, T4A) was analyzed at 10 ppm, high enough to saturate the instrument. In VS (blue) mode, the potential was swept from 280 V/cm to 940 V/cm (6–20 kV applied to the first ring) (blue trace). Using equation [[Disp-formula eq6]], the average field applied for this ion was calculated as 330 V/cm and a static run was performed for direct comparison at the closest value possible for comparison. It is noted that while the drift times remained nearly identical (±0.01 ms), VS mode demonstrated a significantly higher resolving power (68.21) when compared to static (59.79) using the same IMS cell. The slight shift in drift time is due to parasitic capacitance slightly reducing the expected electric field. This can be easily corrected through a one time calibration with a known mobility standard, as discussed with respected to Table 1, below. As these spectra were obtained at the same nominal potential, the signal-noise ratio (S/N) remained constant, though the 2.5 s delay necessitated a significantly longer experimental period for signal averaging.

**Figure 3:**
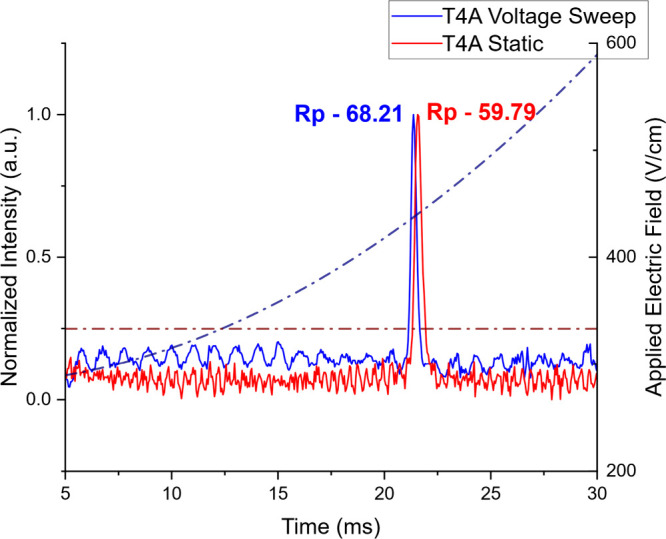
Comparison of static IMS (red) at optimum field applied electric field to voltage sweep IMS (blue). Dashed lines indicate electric field applied to the system for each analysis. VSIMS demonstrated a slightly faster drift time when compared to static due to the changing electric field accelerating the ions during analysis and produced an improved resolving power over static IMS.

**Table 1:** Calculated Average Reduced Mobility for Compounds Investigated with Relative Standard Deviations as Measured for Static (Average Across All Voltages Measured in Triplicate) and VS (Triplicate Data)^a^

Solution	Literature	Static	Sweep
T3A	1.51 ^29,33,34^	1.51 ± 0.2%	1.50 ± 0.1%
T4a	1.28 ^29,33,34^	1.29 ± 0.2%	1.29 ± 0.1%
T5a	1.12 ^29,33,34^	1.12 ± 0.3%	1.12 ± 0.1%
T6a	0.99 ^29,33,34^	0.99 ± 0.3%	0.98 ± 0.1%
Meth	1.64^4^	1.59 ± 0.2%	1.59 ± 0.05%
MDMA	1.47^4^	1.46 ± 0.2%	1.46 ± 0.1%
Heroin	1.05^4^	1.10 ± 0.3%	1.10 ± 0.1%
Cocaine	1.16^4^	1.26 ± 0.2%	1.27 ± 0.1%
Oxycodone	1.17^4^	1.24 ± 0.2%	1.21 ± 0.01%

a All compounds matched within the error of literature values, with the exception of heroin. It is hypothesized that this was due to using air as the drift gas when compared to nitrogen in the literature values.

Figure 4 exhibits the calculated resolving power of each solution for both static and voltage sweep modes (points). Curved traces indicate calculated optimum resolving power for each reduced mobility at each potential (color matched to the experimental data). The two *y*-axes have been adjusted to overlay these data within the same reference frame to adjust for resolving power efficiency as previously described.^20,21^ In static mode, resolving power (R_p_) was measured across a range of applied potentials, from 300 to 1000 V/cm (gate potential) with the measured R_p_ varying widely due to both instrumental theory (Equation [[Disp-formula eq2]]) and decreased signal at low applied potentials obscuring accurate peak measurements. In comparison, VS mode demonstrated an average of 33% higher Rp (p = 3.5e-12) over static mode across all potentials measured. This is likely due to the combination of analyzing each ion at its optimum spectrum and the peak compression effect of accelerating ions during an IMS separation, resulting in later ions experiencing a slightly higher electric field than earlier ions. Interestingly, VS mode resulted in near constant Rp measured across the range of compounds tested, effectively optimizing the separation for the IMS cell regardless of the ion analyzed.

**Figure 4:**
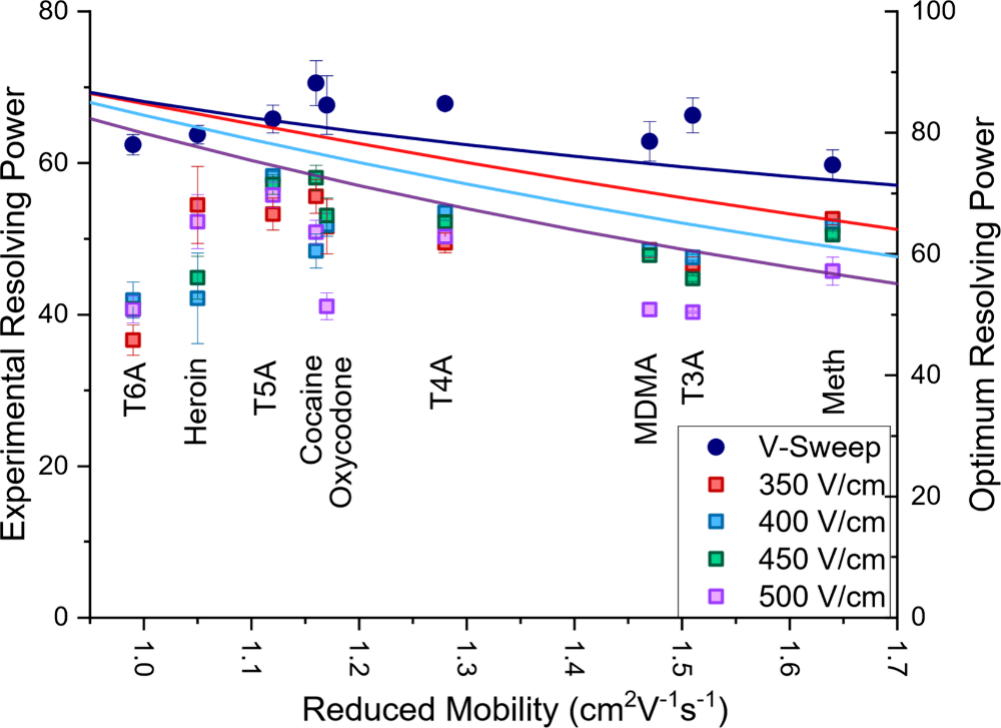
Comparison of measured resolving power in static (at 350, 400, 450, and 500 V/cm) and voltage sweep (300–1000 V/cm sweep) modes. Solid traces indicate theoretical resolving power for the experimental conditions used, with axes adjusted to overlay the data for comparison. VSIMS demonstrated significantly improved and stable resolving powers over static mode at all potentials for all mobilities investigated.

### Mobility Accuracy

Table 1 demonstrates observed mobilities of a series of positive mode standards. The static potential values represent an average mobility calculated from 325, 425, 525, and 625 V/cm electric fields in the drift region and match those observed in the literature for all compounds within ± 0.02 $\frac{cm^{2}}{Vs}$ In voltage sweep mode, equation [[Disp-formula eq6]] provided an average potential for calculating the field strength on the IMS cell for calculating the mobility of the resultant ions. In order to match the reduced mobility values as calculated in the literature, the V_add_ value in equation [[Disp-formula eq6]] had to be adjusted down from the measured value by approximately 12 V/cm. It is hypothesized that this difference is due to the deviation from the ideal potential as demonstrated in Figure 2 and explained above. This was a consistent correction across the breadth of compounds studied, so TH3 was used as a calibration mobility, with the average V_add_ correction calculated using the literature K_0_ of T3A and applying it to all compounds. With the correction, all mobilities matched the static values to within ± 0.35%.

### Mixture

In order to demonstrate the improved resolution and resolving power of the VSIMS method, a mixture of all positive mode samples previously demonstrated was analyzed simultaneously with the results demonstrated in Figure 5. Figures 5a and 5 **b** demonstrate static potential separations at two different applied potentials (00 and 500 V/cm, respectively). At the lower potential, faster ions were better separated, but later ions (especially T5A, heroin, and T6A) reduced signal intensity when compared to the 500 V/cm analysis. However, at 500 V/cm (**5b**), the fast ions separation was reduced, as expected. Figure 5c shows the mixture’s spectrum in voltage sweep mode. In VSIMS mode, all peaks were well separated at relatively equal intensities. The overall signal was reduced in this mode due to the gate depletion effect^33^ under the low field conditions at the initiation of the separation. It should be noted that all spectra demonstrated herein were developed in the positive mode. At the current stage of development, negative mode spectra demonstrate artifacts related to a second triggering of the ion gate electronics caused by the applied voltage sweep. It is hypothesized that a new ion gate controller design would correct this issue and is currently in development. See Supporting Information (Figure S.2) for an example negative mode VSIMS spectrum.

**Figure 5:**
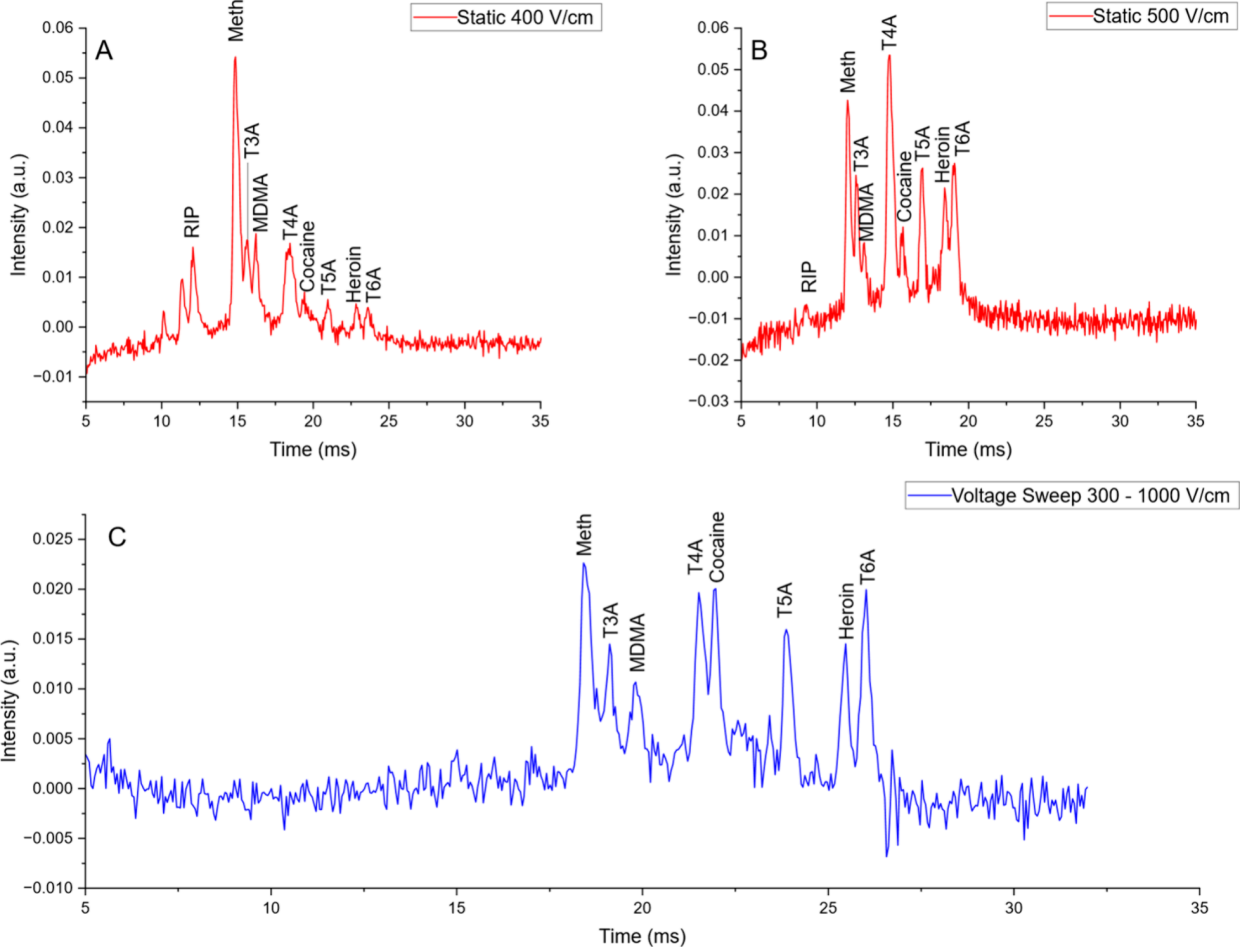
Figure demonstrates a mixture at both static and sweep mode. The mixture is composed of each narcotic at 20 ppm and each TXA salt at 2.5 ppm. (a) The spectrum of the created mixture operated at 400 V/cm in static mode and (b) was operated at 500 V/cm. (c) The blue voltage sweep spectra ran from 300 to 1000 V/cm.

## Conclusions

Established theory of drift tube IMS demonstrates an optimum potential for each separated ion, but is limited by the requirement to establish a single potential at the start of an analysis, thus compromising resolution across the mobility range. Voltage Sweep IMS allows for the modification of the electric field in an atmospheric pressure drift tube IMS cell and significantly improved both resolution and resolving power. At the current stage of development, experimental time is sacrificed in order to allow the associated electronics to stabilize at the starting potential prior to initiating a separation, but further refinement of gate controller electronics should eliminate this additional experimental time. However, unlike alternatives such as Structures for Lossless Ion Manipulations and Cyclic Ion Mobility Spectrometry, VSIMS maintains mobility accuracy without repeated calibration and does not require vacuum conditions in order to function, allowing for small, portable instruments to improve resolution with minimal changes to the operating parameters and no changes to the IMS cell or experiment at atmospheric pressure.

## Supplementary Material





## Notes

Supplemental figures, including a raw VSIMS spectrum of T4A (Figure S1) and initial results in negative mode VSIMS (Figure S2) (PDF)
Python Script – VSIMS control (requires a National Instruments USB or PCI 63xx-based DAQ controller and associated electronics) (ZIP)
