# Supplementary material for: Solving the “General Elution Problem” of Ion Mobility Spectrometry: Single-Run Voltage Sweep
Source: Anal Chem. 2025 Nov 10;97(46):25576–82. doi: 10.1021/acs.analchem.5c04496 (PMC12658867; doi:10.1021/acs.analchem.5c04496)
Supplement: Supplementary file 1 [file ac5c04496_si_001.pdf]

Solving the "General Elution Problem" of Ion Mobility Spectrometry: Single-Run Voltage Sweep

Supporting Information

Addison Bale[1], Tristan Koop[1], Landon Vyhmeister[1], Gavin Valdez[1], Julia Fehr[1], Eric Davis[1*]

[1]Whitworth University, Department of Chemistry, Spokane, WA 99251

[*] Please address all correspondence to Eric Davis (ericdavis@whitworth.edu), Phone: (509) 777-3557

Table of Contents

Table of Contents S.1

Figure S.1 – Raw VSIMS Spectrum S.2

Figure S.2 – Negative Mode VSIMS Spectrum S.3

Description of Additional Supplemental Information Files S.4

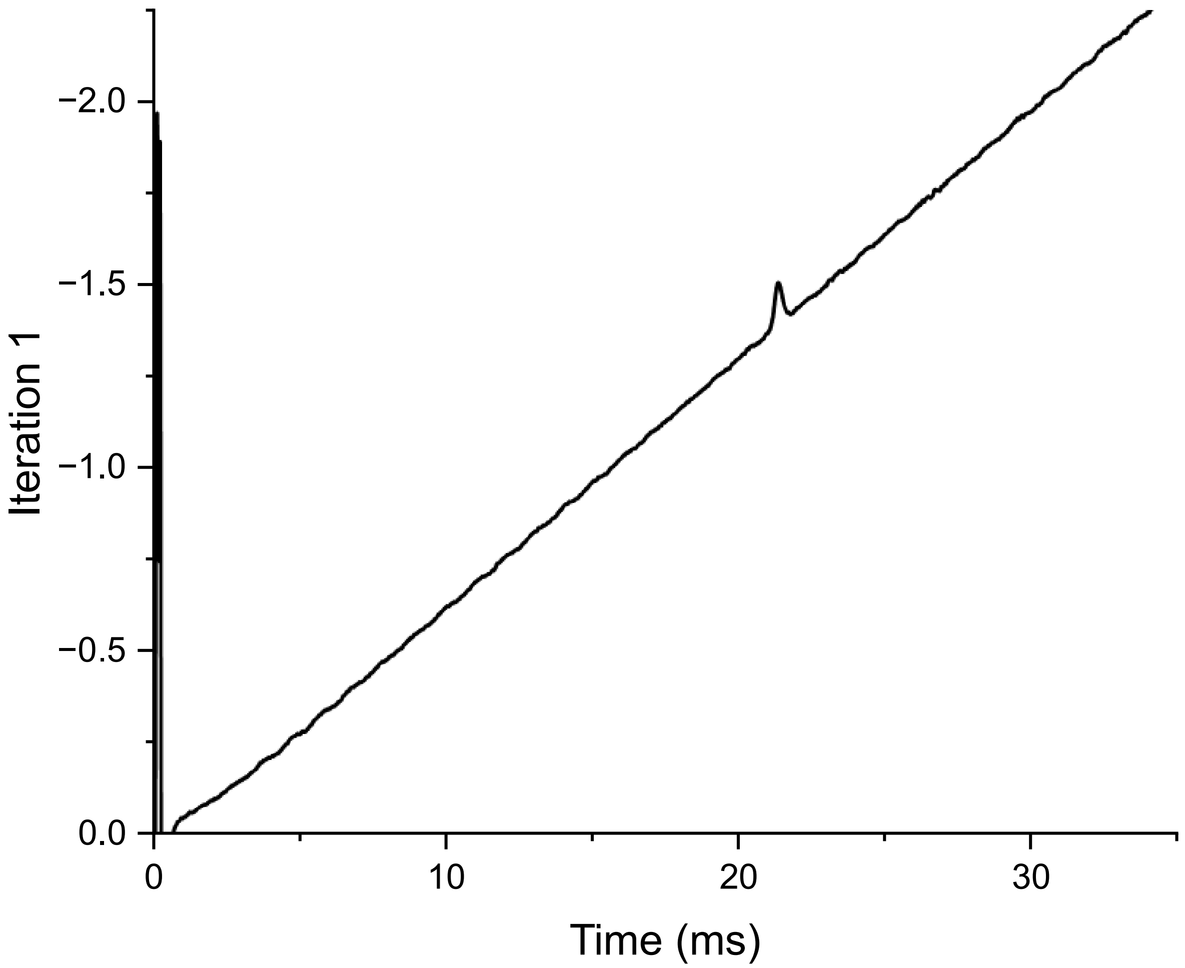

Supplemental Figure S.1: Demonstration raw VSIMS spectrum of T4A. All spectra in manuscript have been baseline corrected to avoid the slope produced through changing electric field in proximity to the Faraday plate detector.

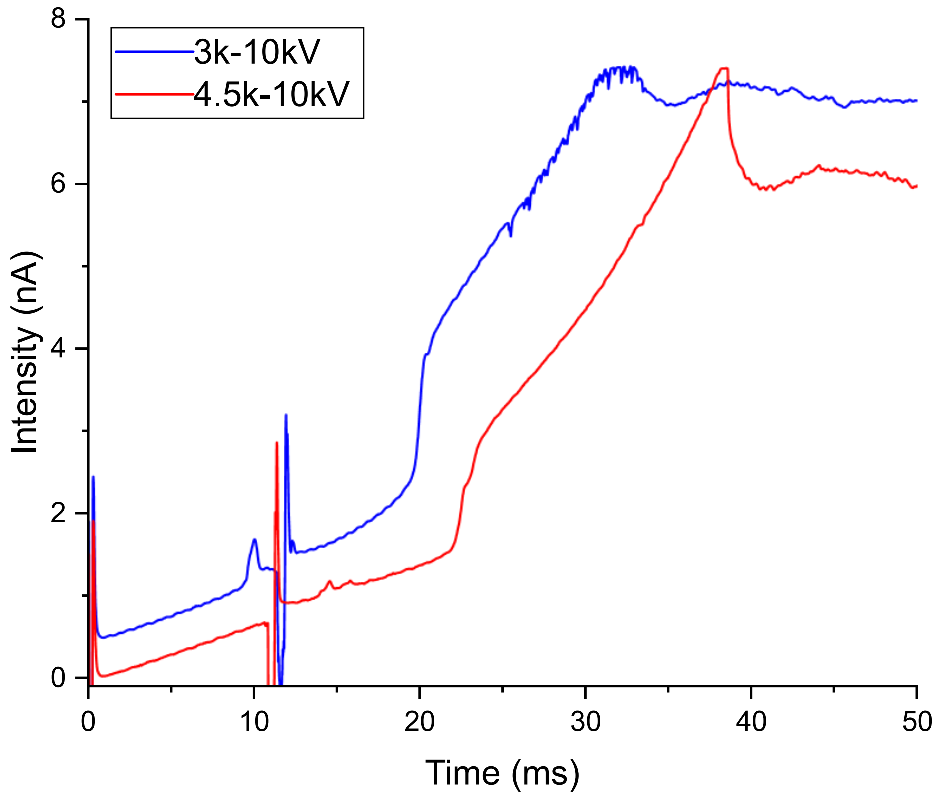

Supplemental Figure S.2: Demonstration of negative mode VSIMS. The artifact noted at ~11 ms was consistent regardless of applied potential. Dual traces indicate changes in start potential and the peaks noted at 10 ms (blue) and 15 ms (red) are analyte peaks demonstrating the voltage dependence of measured mobility. It is hypothesized that the artifact is due to gating electronics and work is in progress to mitigate this effect.

Also Included:

Python Script – VSIMS control. Requires a National Instruments USB or PCI 63xx based DAQ controller and associated electronics.
